# Tuberculose auriculaire concomitante à une localisation pulmonaire chez un patient immunodéprimé par le VIH, à Bamako, Mali

**DOI:** 10.48327/mtsi.v3i4.2023.415

**Published:** 2023-11-17

**Authors:** Farimadiané COULIBALY, Yacouba CISSOKO, Aden Ibrahim BOUH, Djokdelna Ezéchiel GANDAYE, Mariam SOUMARÉ, Issa KONATÉ, Sounkalo DAO

**Affiliations:** 1 Service de Maladies infectieuses et tropicales du Centre hospitalier universitaire du Point G, Bamako, Mali; 2 Faculté de Médecine et d'odontostomatologie de l'Université des Sciences, des techniques et des technologies, Bamako, Mali; 3 Centre de recherche et de formation sur la Tuberculose et le VIH (CEREFO), Bamako, Mali

**Keywords:** VIH, Tuberculose multifocale, Tuberculose auriculaire, Hôpital, Bamako, Mali, Afrique subsaharienne, HIV, Ear tuberculosis, Multifocal tuberculosis, Hospital, Bamako, Mali, Sub-Saharan Africa

## Abstract

**Introduction/Justification:**

La tuberculose demeure un problème de santé publique majeur. Elle constitue une pathologie opportuniste, très fréquente chez les immunodéprimés par le VIH, classant le sida en stade clinique IV de l'OMS en cas de localisations extra pulmonaires. La tuberculose auriculaire reste une forme clinique rare et sous-diagnostiquée. Nous rapportons un cas de tuberculose auriculaire concomitante à une localisation pulmonaire chez un immunodéprimé par le VIH de 32 ans, hospitalisé à Bamako (Mali) afin de discuter des difficultés diagnostiques et thérapeutiques posées par cette localisation rare.

**Description du cas:**

Le patient présentait une toux productive chronique, une otalgie et une otorrhée purulente chronique droite. La recherche de bacilles acido-alcoolo-résistants était positive à l'examen direct dans le liquide de tubage gastrique et l’écouvillonnage du pus auriculaire.

Un traitement antituberculeux institué pendant 6 mois, associé aux adjuvants, a conduit à la guérison complète du patient.

**Discussion/conclusion:**

La localisation auriculaire, bien que rare, doit être activement recherchée. Un traitement étiologique doit être institué pour éviter les complications et les séquelles.

## Abstract (en)

**Introduction/Rationale:**

Tuberculosis remains a major public health issue. It is an opportunistic pathology, very common in HIV-immunocompromised persons, classifying it at the WHO stage 4. Ear tuberculosis remains a rare and under-diagnosed clinical form. We report here a case of ear tuberculosis concomitant with pulmonary localization in an HIV-immunosuppressed person on triple antiretroviral therapy aged 32 years hospitalized in Bamako (Mali) to discuss the diagnostic and therapeutic difficulties posed by this rare localization.

**Description of the case:**

The patient had a chronic productive cough, otalgia and right chronic purulent otorrhea. The search for acid-resistant bacilli was positive for direct examination in gastric casing fluid and swabbing of the ear pus, confirming the diagnosis of tuberculosis. Anti-tuberculosis treatment instituted for 6 months associated with adjuvants resulted in complete healing of the patient.

**Discussion/conclusion:**

Although rare, ear localization must be actively sought. Etiological treatment must be instituted upon confirmation of the diagnosis to avoid complications and sequelae.

## Introduction

La tuberculose (TB) reste un problème majeur de santé publique au niveau mondial [13]. Sa forme auriculaire est très rare, avec moins de 0,1% des otites moyennes chroniques décrites, tandis que la tuberculose pulmonaire représente la forme la plus fréquente, soit 60 *%* des cas [9]. Nous rapportons ici le cas d'une tuberculose de l'oreille moyenne concomitante à une localisation pulmonaire chez un patient immunodéprimé par le VIH. Le but est d'attirer l'attention de nos confrères otorhinolaryngologues, mais surtout généralistes, sur l'otite moyenne chronique tuberculeuse (OMCT) dont la prévalence semble augmenter, de relater les étapes du diagnostic de cette affection rare et d'assurer une prise en charge précoce afin d’éviter les complications entravant le pronostic fonctionnel et vital.

## Cas clinique

Il s'agit d'un patient de 32 ans, boulanger, originaire de Bamako, admis dans le service des Maladies infectieuses et tropicales du CHU du Point G (Bamako, Mali) le 27 avril 2023 pour toux productive chronique, otalgie et une otorrhée purulente chronique droite.

La symptomatologie serait d'installation progressive en 1 mois, initialement traitée dans un centre médical à base d'artésunate, de paracétamol et d'antibiotiques non spécifiés pour paludisme confirmé et otite moyenne aiguë, sans succès. Il est immunodéprimé par une infection à VIH1, diagnostiqué et mis sous trithérapie antirétrovirale (TARV) Ténofovir/Lamivudine/Dolutégravir il y a 7 mois, inobservant pour raison du déni de sa maladie.

L'examen physique général retrouve une fièvre (38,2 °C), une altération de l’état général, une otalgie, une otorrhée purulente fétide droite, un syndrome de condensation pulmonaire basal droit, un examen neurologique normal, sans l'atteinte des nerfs crâniens principalement du nerf facial VII et du nerf cochléovestibulaire VIII.

**Figure 1:**
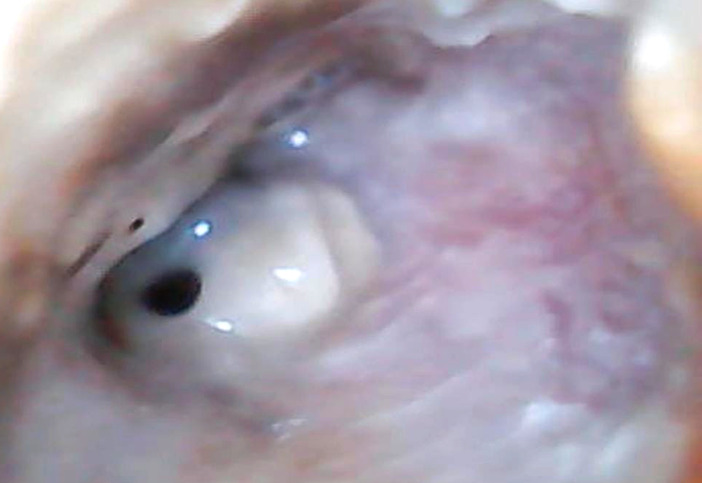
Otoscopie de l'oreille droite avant traitement: conduit auditif externe inflammatoire avec des sécrétions purulentes et présence d'une perforation tympanique unique dans le cadran antéro-inférieur Otoscopy of the right ear before treatment: inflammatory external auditory canal with purulent secretions, and presence of a unique tympanic perforation in the anterior-inferior dial

À l'examen ORL, l'otoscopie de l'oreille droite objective un conduit auditif externe inflammatoire avec des sécrétions purulentes et la présence d'une perforation tympanique unique dans le cadran antéro-inférieur (Fig. 1). L'oreille gauche est normale. L’épreuve de Rinne et Weber est en faveur d'une surdité de transmission droite.

L’évaluation immuno-virologique relève un taux de CD4 à 118 cellules/pl et une charge virale à 12 370 copies/ml au moment du diagnostic de l'infection par le VIH contre une charge virale à 9 460 copies/ml et un taux de lymphocytes CD4 à 193 cellules/pl au 6^e^ mois sous traitement antirétroviral. Au moment du diagnostic de la tuberculose au 7^e^ mois, l’évaluation immuno-virologique objective un taux de CD4 à 89 cellules/pl et une charge virale à 10 230 copies/ml.

La bacilloscopie après coloration au Ziehl Neelsen est positive à une croix dans le liquide de tubage gastrique dès l'admission et 19 jours plus tard dans l’écouvillonnage du pus de l'oreille droite vu la persistance de l'otorrhée. Le test Xpert-MTB/GeneXpert ne détecte pas de *Mycobacterium tuberculosis* résistant à la rifampicine.

**Figure 2:**
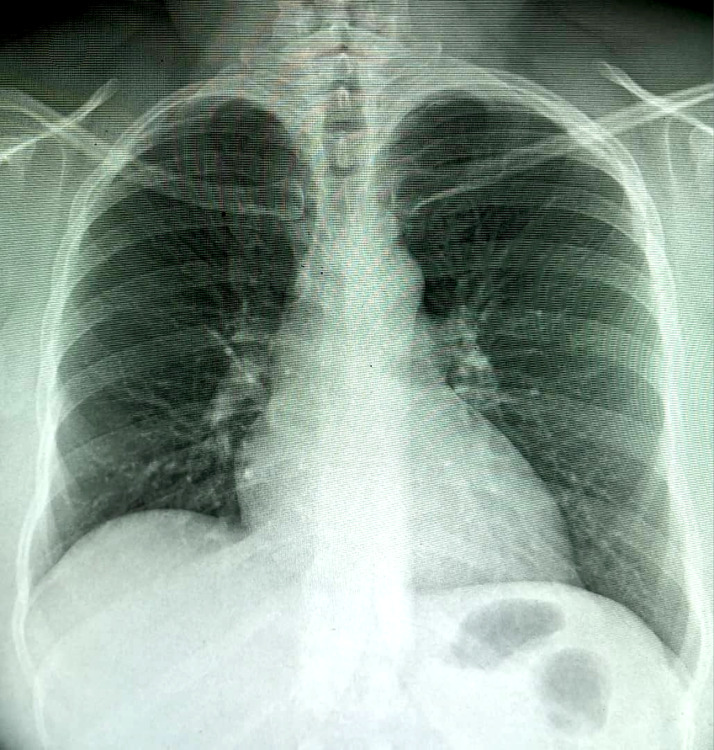
Radiographie du thorax de face: accentuation de la trame bronchovasculaire surtout au niveau de la base pulmonaire droite et cardiomégalie modérée avec index cardiothoracique à 0,60 Frontal chest x-ray: accentuation of the bronchovascular pattern more at the level of the right pulmonary base and moderate cardiomegaly with cardiothoracic ratio at 0.60

La radiographie thoracique de face montre une accentuation de la trame bronchovasculaire plus accentuée au niveau de la base pulmonaire droite (Fig. 2).

Le diagnostic de tuberculose de l'oreille moyenne concomitante à une localisation pulmonaire sur le terrain d'immunodépression par le VIH1 est donc retenu.

Le patient est mis sous antituberculeux oraux de première ligne pour une durée de 6 mois, à dose fixe d'une quadrithérapie en phase intensive d'isoniazide, rifampicine, pyrazinamide et d’éthambutol durant 2 mois (2RHZE), suivie d'une bithérapie en phase d'entretien d'isoniazide et de rifampicine durant 4 mois (4RH) à raison de 3 comprimés/jour le matin à jeun, associés à la vitamine B6 (1 comprimé/jour). Il bénéficie de deux séances de renforcement thérapeutique aux antirétroviraux. Le TARV est réinitié le 4 mai 2023 avec son consentement, vu la bonne tolérance des antituberculeux, par la combinaison Ténofovir/Lamivudine/Dolutégravir à raison d’1 comprimé/jour associé à du Dolutégravir 50 mg en supplément (1 comprimé/jour) selon le protocole de prise en charge du VIH/sida. Une chimioprophylaxie au cotrimoxazole 960 mg (1 comprimé/jour) est entreprise ainsi qu'un nettoyage du conduit auditif externe par aspiration et l'instillation de ciprofloxacine goutte auriculaire (2 gouttes 3 fois par jour) pendant 14 jours.

L’évolution est favorable à 14 jours sous traitement, marquée par une apyrexie, un bon état général, l'amendement de la toux et de l'otorrhée avec la négativation de la bacilloscopie dans le liquide du tubage gastrique et l’écouvillonnage du pus de l'oreille droite. À la fin du traitement antituberculeux d'entretien, la guérison clinique est complète avec un examen ORL et neurologique normal. La bacilloscopie associée au test Xpert-MTB/GeneXpert à la fin du 2^e^ mois de la phase intensive, au cours du 5^e^ mois et à la fin du 6^e^ mois de la phase d'entretien sont revenus négatifs. La charge virale VIH réalisée au bout de 3 mois de TARV est de 329 copies/ml.

## Discussion

La tuberculose diffuse ou multifocale représente dans la littérature 9 à 10% des cas de tuberculose extra-pulmonaire [5, 6]. L'otite tuberculeuse est une étiologie rare de l'otorrhée, ainsi, c'est un diagnostic rarement évoqué devant une otite moyenne subaiguë ou chronique [2, 7]. L'otite moyenne tuberculeuse doit être fortement suspectée chez les patients tuberculeux présentant une infection chronique de l'oreille. L'atteinte est le plus souvent unilatérale [1, 3], comme dans notre cas.

La contamination de l'oreille moyenne se fait directement à partir des poumons, *via* le larynx, le nez et la trompe auditive, par voie sanguine ou par inoculation directe à travers une perforation tympanique [8, 12].

La triade classique de la tuberculose auriculaire associant une otorrhée, une paralysie du nerf facial et des perforations tympaniques multiples « pomme d'arrosoir » est rarement observée [7]. Chez notre patient, les signes cliniques (l'otorrhée purulente chronique, une surdité de transmission droite) et les aspects otoscopiques (la présence de sécrétions purulentes et de perforation tympanique) ont fait évoquer les diagnostics différentiels suivants: otite moyenne chronique suppurative, otite moyenne chronique cholestéatomateuse.

L'atteinte pulmonaire associée facilite le diagnostic de la tuberculose auriculaire [14]. Dans notre cas, la tuberculose pulmonaire a été diagnostiquée en premier et la forme auriculaire a été confirmée 19 jours plus tard devant la persistance de l'otorrhée. Ce retard du diagnostic de la tuberculose auriculaire s'explique par la localisation rare, ce qui soulève des difficultés diagnostiques, car elle peut simuler longtemps une banale otite chronique.

Les examens bactériologiques ou anatomopathologiques de biopsie ou pièce opératoire confortent le diagnostic de la tuberculose de l'oreille moyenne, avec une sensibilité de 70 à 90% [11]. Le prélèvement de sécrétions auriculaires et la recherche de bacilles acido-alcoolo-résistants (BAAR) sont d'intérêt capital dans le diagnostic définitif de l'otite tuberculeuse. Or, la présence des BAAR dans des produits pathologiques autres que dans les crachats à l'examen direct est rare, à cause du nombre insuffisant de *Mycobacterium tuberculosis* dans ces autres localisations [12]. La tomodensitométrie à haute résolution est l'examen de référence pour apprécier l'extension [12].

L’évolution est souvent défavorable avec complications, voire décès possible. Sinon des séquelles motrices, faciales et auditives sont fréquentes [10]. Dans notre cas, l’évolution était favorable avec un examen ORL et neurologique normal à la fin du traitement.

Le traitement médicamenteux repose sur les antituberculeux durant 6 à 9 mois. La chirurgie est indiquée à visée diagnostique, afin de prélever des tissus pathologiques pour des examens histologiques et bactériologiques ou en seconde intention, en cas d’échec du traitement médical [4]. Dans notre cas, le traitement anti-bacillaire était conforme aux recommandations du Programme national de lutte contre la tuberculose au Mali pour une durée de 6 mois, permettant une guérison complète.

## Conclusion

L'otite tuberculeuse est une forme rare, qui mérite d’être connue, afin de préserver le pronostic fonctionnel de l'oreille et d’éviter les complications irréversibles.

Le diagnostic de l'otite tuberculeuse doit être suspecté devant toute symptomatologie traînante de l'oreille, résistante au traitement habituel et particulièrement dans les populations à risque.

## Consentement éclairé

Notre patient a donné son consentement éclairé pour la publication de son dossier médical sous anonymat.

**Figure 3:**
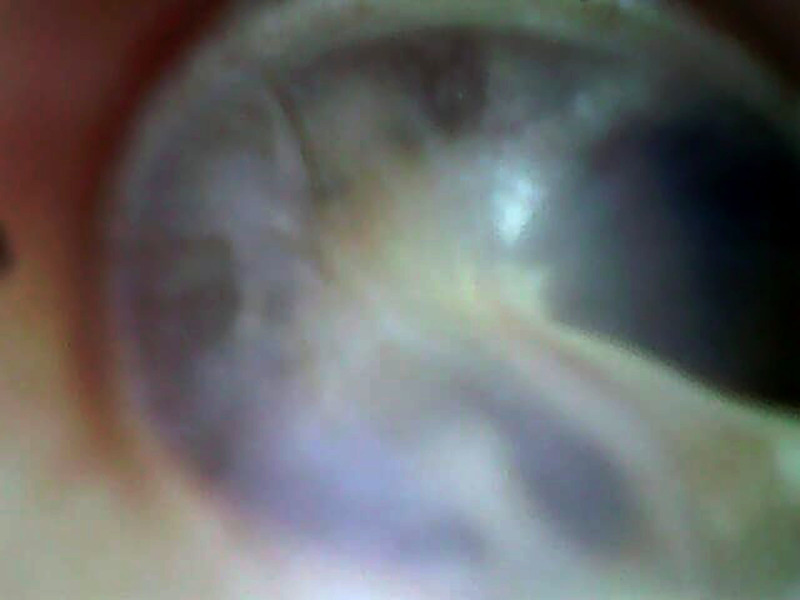
Otoscopie de l'oreille droite après traitement: conduit autidif externe normal et tympan normal de transparence grisée avec visibilité des reliefs de la caisse tympanique et de la cicatrice Otoscopy of the right ear after treatment: normal external auditory canal, and normal tympanum of gray transparency with visibility of the reliefs of the tympanic body and the scar

## Contributions des auteurs

Farimadiané COULIBALY: Conception du cas clinique, prise en charge du patient, revue de la littérature, rédaction du manuscrit. Aden Ibrahim BOUH, Djokdelna Ezéchiel GANDAYE: Prise en charge du patient, revue de la littérature, apport critique, approbation de la version finale à publier. Yacouba CISSOKO, Mariam SOUMARÉ, Issa KONATÉ, Sounkalo DAO: Prise en charge du patient, apport critique, correction du manuscrit et approbation de la version finale à publier.

## Conflits d'intérêts

Les auteurs ne déclarent aucun lien d'intérêts.
